# An Assessment of Fabrication, Properties, and Medical Applications of Chitosan–Nanometal Coatings

**DOI:** 10.3390/ma18235322

**Published:** 2025-11-26

**Authors:** Michał Bartmański

**Affiliations:** Department of Biomaterials Technology, Faculty of Mechanical Engineering and Ship Technology, Gdańsk University of Technology, 80-233 Gdańsk, Poland; michal.bartmanski@pg.edu.pl; Tel.: +48-500-034-220

**Keywords:** chitosan-based coatings, metallic nanoparticles (NPs), antibacterial properties, deposition methods, noble and semi-noble metals (Au, Ag, Cu, Zn)

## Abstract

Chitosan (chit) is a specific polymer, an oligosaccharide, that demonstrates several important properties alone or in combination with other compounds or elements. These important properties include being biocompatible with the human body and simultaneously effective in destroying bacteria. Moreover, it is a smart polymer, as it can change its properties when the pH value decreases below about 7. Therefore, chitosan is widely applied in dermo-cosmetics, but it is also intensively investigated for its use in food preservation and the targeted regeneration of teeth in dentistry. Due to these advantageous characteristics, chitosan has been employed in numerous coating systems for biomedical applications. These coatings can be fabricated through a wide variety of procedures involving different deposition techniques, process parameters, and the chemical characteristics of the environment, solution, air or vacuum, as well as the substrate. Chitosan–metallic coatings have often been investigated; however, the use of elementary metals in the form of nanoparticles (NPs) was rarely reported. The main part of this paper is focused on the presentation of chitosan–metallic NPs, in particular, an application of potentially antibacterial noble and semi-noble metals such as Au, Ag, Cu, and Zn, intensively investigated by the author. The deposition methods and their limitations, the differences in properties of such coatings and those possessing Ag, Sr, Zn, and other metals in the form of cations in chemical compounds, and the perspectives of chit–MeNPs (metal nanoparticles) are thoroughly considered, and future research is proposed.

## 1. Characteristics, Properties, and Applications of Chitosan

Chitosan (chit), the deacetylated derivative of chitin, is a specific polymer, classified as an oligosaccharide, that demonstrates several important properties alone or accompanied by other compounds or elements. Among them, chitosan is wholly biocompatible, and it has some antibacterial efficiency. Chitosan is soluble in slightly acidic environments due to the presence of the amino groups, and it is insoluble in neutral and alkaline media, so it might be assessed as a smart polymer. Moreover, its coatings are characterized by remarkable adhesion and mechanical behavior. As equally important, chitosan promotes osseointegration, demonstrated by cell adhesion, viability, and proliferation, as well as improved mineralization. Its chemical structure can be modified due to the presence of amine and hydroxyl groups, allowing for the incorporation of nanomaterials and improving its mechanical and biological appearance [1,2,3]. It can be derived from different sources, such as crustacean shells, fungi, and microalgae [4].

In addition to these well-established physicochemical and biological properties, recent literature highlights that the performance of chitosan can be further enhanced through blending with other natural biopolymers, such as starch. Several studies, including the work presented by Oyekunle et al. [5], demonstrate that the incorporation of starch improves the mechanical stability, barrier performance, and overall quality of chitosan-based coatings and composite materials. This synergistic behavior arises from the increased availability of hydrogen-bonding sites and improved structural organization within the blended polymer matrix. Such enhancements are particularly relevant for chitosan–nanometal systems, where the integrity and functionality of the polymer matrix directly influence the uniformity, stability, and biological response of the resulting materials.

Various methods are used to deposit chitosan coatings. They include electrophoretic deposition (EPD) [6], layer-by-layer (LBL) technique, solution casting, and dip and spin coating [1]. The recent investigations included pulse electrodeposition of CaP (calcium phosphate)–chit [7], constant voltage electrodeposition of carboxylic acid/hydroxyapatite (HAp)/chit coatings [8], and chitosan/Eudragit E100 or poly(4-vinylpyridine)/mesoporous bioglass coatings made by EPD [9]. It can be successively applied for an alloy produced by selective laser melting and forging [10]. It is also worth noting its application together with calcium phosphate as a coating on biodegradable Mg alloy [11]. Although a variety of fabrication techniques have been successfully used to prepare chitosan–nanometal coatings, each has its intrinsic limitations that should be taken into consideration. Although EPD is versatile and suitable for complex geometries, the process may result in non-uniform nanometal distribution, agglomeration, and internal stresses, particularly at higher voltages, compromising coating integrity and adhesion. LBL assembly allows excellent control over the thickness and composition; however, it is labor-intensive and may be difficult to scale up for large or irregularly shaped implants. Additionally, simple methods such as dip-coating or spin-coating generally offer relatively thin layers that have limited loading capacity and may be sensitive to solution stability and drying conditions.

Chitosan can be processed as nanoparticles, nano-vehicles and nanocapsules, fiber meshes and scaffolds, and 3D-printed scaffolds for a variety of applications [12]. In particular, nanoparticles introduced into chitosan matrices improve their bioactivity, as cells tend to proliferate and differentiate faster than in the bulk or micron-sized scaffolds. The applications of chitosan and chit-based materials include mainly regenerative medicine, wound dressings, the delivery of bioactive agents such as genes, drugs, and proteins, cancer treatment, and as a part of organs-on-chips, and biosensing applications [12,13]. Chitosan-based biomaterials were considered in the treatment of bone disorders [14], as a self-lubricating antibacterial hydrogel on titanium joint implants [15], chitosan hydrogel with mesoporous silica nanoparticles, and ibuprofen as a release system for surface coating of titanium implants [16]. For better bone regeneration, chitosan-based scaffolds have been used with several growth factors, such as periostin and osteopontin, to initiate osteogenesis and angiogenesis and increase cell proliferation [17]. It can form a coating of elastomeric orthodontic modules [18]. Non-biofouling properties appear against fluorescent-tagged proteins such as bovine serum albumin [19]. Quaternized chitosan/glycyrrhizic acid co-decorated titanium with enhanced antimicrobial, immunomodulatory, and osteogenic properties was recommended for dental implant applications [20]. The bioactive and antibacterial coatings based on silica, gentamicin, and chitosan were proposed to improve the early-stage performance of titanium implants [21].

Antibacterial behavior can be achieved in medical applications using different solutions. For example, antibiotics such as chlorhexidine-loaded [22] mesoporous polydopamine, levofloxacin [23], and amikacin [24] are used for antibacterial activity. In [25], such a role is played by black phosphorus deposited on HAp and chitosan layers to enhance both antibacterial activity and osseointegration. The antibacterial properties against *S. aureus* and *E. coli* of some polymer composite materials with the addition of CeO_2_ have been reported [26]. However, nanometals, such as noble Ag and, less often, Au, semi-noble Cu, Zn, and occasionally other metals, have been implemented to make the interface of titanium or stainless steel antibacterial [27], and these additions are the subject of this review as the most promising options. Derived from sources such as plants, microbes, and waste green solvents, green nanometals (GNMSs) or semiconductors (GNSSs) were obtained, including ZnO, TiO_2_, SnO_2_, and C_3_N_4_, demonstrating photocatalytic and ROS (reactive oxygen scavenger)-generating capabilities whereas GNMSs, like silver, copper, and gold, exhibited antimicrobial properties by ion release and membrane disruption [28]. Also, Ag, ZnO, and Pt were indicated as particularly antibacterial nanomaterials [29]. Interestingly, a novel eco-friendly method for synthesizing silver nanoparticle (AgNP)-decorated chitosan film by sucrose hydrolysis with high antibacterial efficacy has been proposed [30].

Chitosan can also be used in environmental protection. For example, the carboxylated chitosan–phthalate/ZrO_2_ nanocomposite material is capable of removing methylene blue dye [31]. The chitosan-coated date palm endocarp film stabilized with silver nanoparticles enhances the catalytic reduction of p-nitrophenol [32].

The antibacterial effect of chitosan-based composite materials and coatings was also successfully applied in other domains. The most powerful seems to be used in food technology, in particular, for chitosan obtained by green synthesis. As demonstrated in [33], different metallic nanoparticles can be used for the production of food packaging materials, increasing the shelf life of packaged foods, and maintaining food quality. As of today, the most prevalent NPs are represented by ZnO, TiO_2_, and CuO. Additionally, as smart food packaging materials are cellulose-based composites containing AgNPs, they change their color with the release of volatile components from fish and meat. Another example is alginate with AuNPs, which changes with storage temperature and duration. AgNPs and CuONPs are applied because of their strong antimicrobial properties, and the AuNPs and MgONPs are used as substances that are reactive under UV light exposure. TiO_2_NPs and ZnONPs are photocatalytically active, enhancing the antimicrobial barrier under UV exposure. Chitosan with nanoAg seems especially important for food preservation against bacteria and fungi [34,35,36,37,38,39,40].

**Figure 1:**
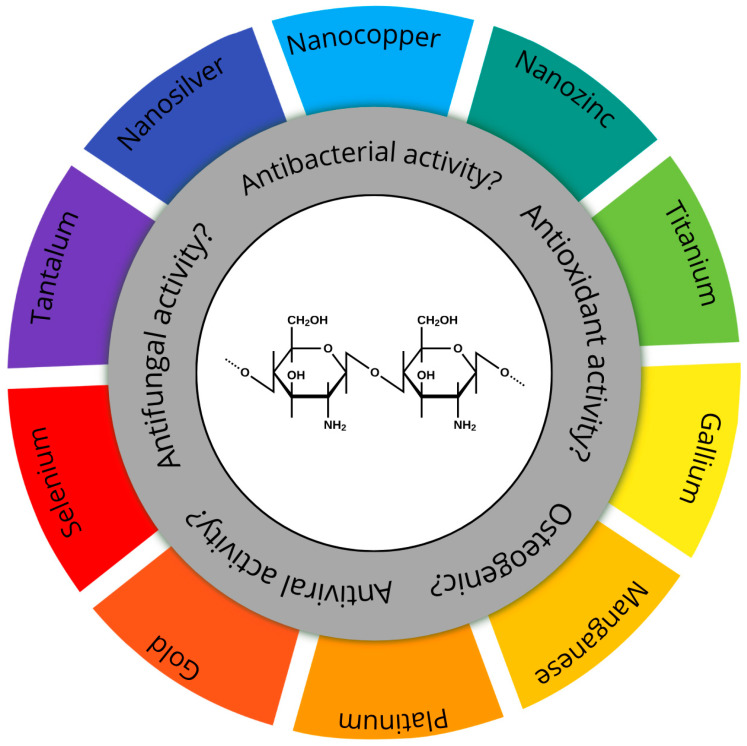
Schematic representation of potential biological properties of chitosan-based composite coatings containing various metallic nanoparticles. The central part depicts the chemical structure of chitosan.

This review encompasses publications from 2013 to 2025 retrieved from Scopus, Web of Science, PubMed, MDPI, and Google Scholar databases. The search strategy included the following keywords: ‘chitosan coatings’, ‘metal nanoparticles’, ‘chitosan-Ag’, ‘chitosan-Cu’, ‘chitosan-Zn’, ‘biomedical coatings’, and ‘antibacterial nanocomposites’. The analysis covers peer-reviewed original articles as well as selected review papers. This review aims to present the key properties of chitosan-based composite coatings, with a special focus on their relevance and functional behavior in biomedical applications (Figure 1).

## 2. Chitosan–Nanosilver Coatings and Composite Materials

**Figure 2:**
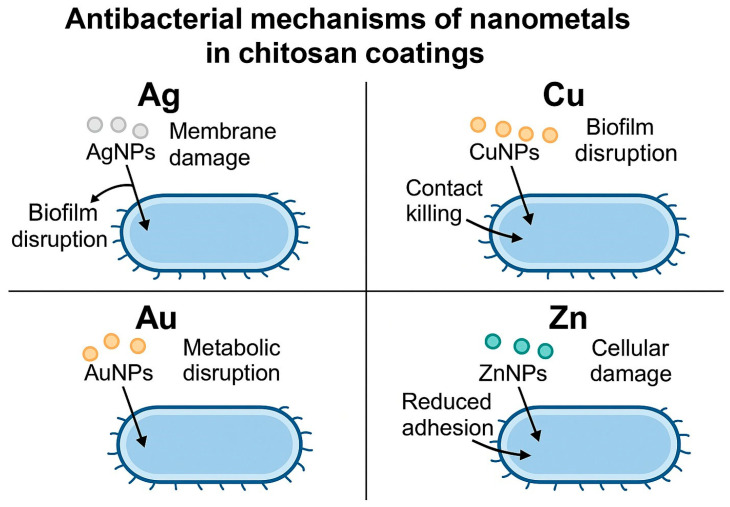
The schematic representation of the proposed antibacterial mechanism of chitosan–AgNPs, chit–CuNPs, chit–AuNPs, and chit–ZnNPs coatings. The illustration is the author’s own elaboration.

The enrichment of chitosan with nanosilver is quite often found in medical applications, in different forms and medical domains, and also on TiO_2_ layers [41]. Bartmański et al. [42] proved its antibacterial properties against *E. coli* and *S. aureus* and that it is sustainable for a certain time due to AgNPs’ presence. The silver-containing chitosan coatings exhibited a significantly high level of antibacterial activity, which was evinced by a substantial reduction in viable *E. coli* and *S. aureus* cell numbers. The enhanced bactericidal effect is attributed to the capacity of AgNPs to disrupt bacterial cell envelope integrity, reflected in the reported degradation of membrane structures and the disruption of biofilm organization. Besides that, the cationic character of chitosan could provide good electrostatic interaction with negatively charged bacterial surfaces, facilitating closer contact between AgNPs and the cell membrane. This synergistic interface facilitates membrane destabilization, leakage of intracellular components, and inhibition of biofilm development, and thus elicits a remarkably stronger antibacterial response compared to chitosan coatings without AgNPs. The schematic representation of the proposed antibacterial mechanism of chitosan–AgNP coatings (and subsequently described chit–CuNP, chit–AuNP, and chit–ZnNP systems) is presented in Figure 2.

It is interesting to note a novel biomimetic technology strategy to obtain chitosan-based composite material [43] based on the use of chit–DHBA (3,4-dihydroxybenzaldehyde) as a reducing, capping, dispersing, charging, and film-forming compound for the reduction of silver ions, their bonding to the composite base, and its EPD. Hileuskaya et al. [44] found that chitosan–Ag was revealed to have remarkable antioxidant activity. Devi et al. [45] reported that cationic carboxymethyl chitosan nanofibers joined with Ag showed enhanced antibacterial applications. Önal et al. [46] proved that Zn (II)/Pd (II) porphyrin-immobilized chitosan hydrogels with silver nanoparticles were effective in photodynamic antimicrobial therapy. For Ag-powered no-chitosan coatings, Jia et al. [47] demonstrated an excellent corrosion protection and germicidal effect against *S. aureus* with AgNPs immobilized onto the surface and incorporated into the bulk cavities of a micro/nanoporous TiO_2_ ceramic coating with no chitosan. Thermochemical treatment resulting in an Ag-doped calcium titanate layer on implants inhibited the biofilm formation with no significant effect on the behavior of osteoblasts [48].

Chitosan-coated silver nanoparticles were proposed against SARS-CoV-2 [49]; 3% Ag@ZnO-loaded films on chitosan/PVA film demonstrated inhibition zones of 21.4 mm for *S. aureus* and 23.2 mm for *E. coli*, inhibition of biofilm formation by 84.3% for *S. aureus* and 86.1% for *E. coli*, and significant anticancer potential for ±HepG2 cells [50]. A similar solution [51] based on PVA-capped AgNPs in a chitosan matrix showed bactericidal activity against *E. coli* and *S. aureus* for 8 h due to the slow release of silver ions. Electrochemically deposited chitosan–Ag complex coatings on biomedical NiTi alloy for antibacterial application were reported in [52]. For the same alloy, the Ag additive to the bioceramic coating significantly increased antibacterial properties but caused a decrease in cell viability (*Saos-2* osteoblast cells) [53]. Marsico et al. [54] reported that a nanocomposite of Ag–chitosan tested against two bacterial strains, *Escherichia coli* and *Micrococcus flavus*, showed marked antimicrobial activity against *E. coli* up to 0.006 g/L, at which value AgNPs alone lost their activity. The antibacterial tests against *B. subtilis* and *E. coli* revealed that the chitosan film decorated with AgNPs reported in [30] exhibited markedly higher efficacy than its precursors and was comparable or even superior to standard controls (amoxicillin and betadine). The carboxymethyl chitosan-immobilized silver nanoparticles for intravesical instillation were claimed as a strategy to prevent catheter-associated urinary tract infections [55].

Nawaz et al. [56] found that chitosan/gelatin/Ag–MnNP-doped mesoporous bioactive glass on the PEEK/bioactive glass layer was formed by EPD on 316 L stainless steel for orthopedic applications, resulting in a strong inhibition of the bacterial colonization with a negligible toxic effect.

The carboxymethyl–chitosan and chitosan were also discussed as bioactive delivery systems by Alemi et al. [57]. In [58], a carboxymethyl chitosan–silver–ginger nanocomposite showed high antibacterial efficacy against *S. aureus* and *E. coli* and high antimicrobial efficacy against *Aspergillus niger* and *Candida albicans* compared to chitosan alone.

Anitha et al. [59] synthesized chitosan-coated AgNPs using Moringa oleifera flower extract against triple-negative breast cancer. In [60], Sericin–chitosan implemented with AgNPs protects against 1,2-dimethylhydrazine-induced colon cancer in mice. In [61], a Schiff base of chitosan prepared by condensing chitosan with aromatic aldehydes was supported with polyvinyl alcohol (PVA), and silver/silver-doped bioactive glass was observed to possess strong antimicrobial activity. Castillejo et al. [62] mixed chitosan with tripolyphosphate (TPP), nanoHAp, and nanosilver, and other supplementary substances to make a hydrogel. The porous titanium was either infiltrated with the hydrogel or covered with it by an immersion method. The antibacterial activity was tested with three different strains, including Gram+ and Gram-negative bacteria, demonstrating excellent inhibition after 21 days.

In dentistry, Amaechi et al. [63] showed that chitosan-based nanosilver fluoride (CNSF) influenced the shear bond strength of restorative materials: it did not significantly affect composite bonding, but it reduced the shear bond strength of resin-modified glass ionomer, likely due to interactions between chitosan and the RMGI setting components.

Then, in dressing wounds, lignin-wrapped silver-implemented chitosan–polyvinylpyrrolidone nanocomposite hydrogel films were applied to infected wounds [64]. PVA–chit biofilms doped with tea tree oil and enhanced with AgNPs can be used in wound dressing applications [65]. Karatas et al. [65] indicate that the presence of tea tree oil increases the degree of cross-linking within the polymer matrix, affecting hydrogen bonding and nanoparticle dispersion in the composite, while simultaneously reducing the swelling ratio and enhancing hydrophobicity as the TTO concentration increases. The haemostatic and antibacterial wound dressing was developed based on chitosan–silk fibroin composite with AgNPs and deferoxamine [66]. Gholizadeh et al. [67] proposed chitosan reinforced with mesoporous silica NPs and silver NPs for the treatment of diabetic wound healing. Thinakaran et al. [68] proved that chitosan, together with high molecular weight PEG (polyethylene glycol) and AgNPs, could be used as a wound dressing material. The chitosan nanoparticles mixed with nitrogen ionic gelation with sodium tripolyphosphate and loading with silver ions enhanced the antibacterial property against *Staphylococcus aureus*, the number of bacteria being 50 to 500 times less, as compared to bulk chitosan, and recommended to use in the fabrication of textiles.

In [69], thiolated chitosan-stabilized silver nanoparticles functionalized with anti-Leishmania infantum antibodies were evaluated to evaluate the diagnostic of visceral leishmaniasis (tropical disease) through an optical biosensor.

Regarding catalytic properties of NPs, AgNPs placed on chitosan–glutaraldehyde polymers were efficient as a catalyst for the synthesis of propargylamines [70]. Chi–Ag was used for the removal of cadmium ions [71].

In food technology, it was proposed jointly with nanosilver to protect lemon from green mold [34] with bentonite and gallotannin and nanoAg for different food preservation [35], as a film of chitosan–flavonoid–AgNPs for shelf life extension of food [36], AgNPs for fresh food preservation [37,38], such as PVA–chit with tea tree oil and AgNPs can be used for food packaging [65]. Also, chitosan coatings with zinc oxide and silverAg–ZnO microspheres were quite effective against spoilage bacteria of aquatic products, *Shewanella putrefaciens*, and *Pseudomonas aeruginosa* [72].

## 3. Chitosan–Nanocopper Coatings and Composite Materials

Copper was attempted to be used in ionic form in medicine. As described by [73], a metal–organic framework with copper ions demonstrated the release of Cu^2+^ ions over 21 days. In vitro experiments showed that such a coating, designed for the nerve system, promoted differentiation and spreading of Schwann cells. In [74], chitosan–CuNP coating was created by EPD on a TiO_2_ nanotubular oxide layer of Ti13Zr13Nb alloy. In [42], some moderate antibacterial properties against *E. coli* and *S. aureus* were shown. Chitosan–copper composite coatings generally exhibit less intense, but measurable, antibacterial properties compared with Ag-containing systems. The antimicrobial action of copper-modified coatings is generally mediated at the surface and impairs bacterial adhesion and the integrity of the biofilm. The experimentally confirmed release of copper ions from the coatings in [42] supports the assumption that ionic copper contributes to bacterial growth inhibition. These contact-dependent interactions, taken together with the intrinsic properties of copper as an antimicrobial agent, act to reduce bacterial adhesion and viability, both for Gram-negative and Gram-positive strains. The schematic representation of the proposed antibacterial mechanism of chitosan–CuNP coatings is presented in Figure 2.

Copper oxide was also the aim of research. Liu et al. [75] studied the importance of copper by the use of Cu-bearing titanium alloy with a hierarchical structure. The TiCu alloy surfaces resulted in antibacterial trapping characteristics due to CuO present in the outermost oxide layer and optimal osteogenicity. In the osteomyelitis-modeled mice, TiCu surfaces significantly prevented the infection and increased the formation of new bone around the implants. In other work [76], deposition of copper-doped tannic acid on porous titanium dioxide resulted in high antibacterial adhesion properties and antibacterial rates for *S. aureus* and *E. coli* of 95.27% and 97.19%, respectively. In research by Ayala-Peña et al. [77], a sprayable chitosan-based solution embedded with copper oxide NPs demonstrated antibacterial activity against both Gram-positive and Gram-negative bacteria, as well as immediate activity against a wide range of viruses. In this context, several works have investigated the role of metallic nanoparticles as antimicrobial agents. Other known studies were aimed at food storage. El-Wahab et al. [78] demonstrated the importance of Co and Cu nanoparticles in fungicidal preservation.

## 4. Chitosan–Nanozinc Coatings and Composites

**Figure 3:**
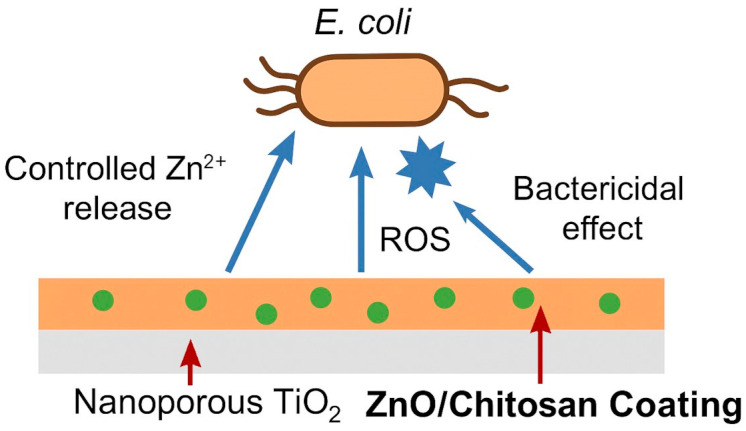
Schematic illustration of the antibacterial mechanism of the ZnO/chitosan coating deposited on nanoporous TiO_2_. The illustration is the author’s own elaboration.

Elementary zinc, capable of forming zinc ions, is seldom proposed as a part of coatings or composites. The silane-coupled chitosan–cyclodextrin/rosmarinic acid–zinc coating improved the osseointegration of titanium implants [79]. Gelatin hydrogel containing zinc ions and polydopamine was observed to kill bacteria and promote osteogenic differentiation of bone marrow stem cells [80]. Another zinc compound, zinc carbonate NPs with accompanying chitosan, cellulose, and hyaluronic acid, was proposed for several medical applications [81]. Finally, chitosan functionalized with lanthanum–zinc ferrite NPs demonstrated hemocompatibility and antibacterial properties [82]. Bartmański et al. [83] reported the presence of zinc NPs; however, cytotoxicity was observed, presumably due to the high metal content. Similarly, Bartmański et al. [84] also noted cytotoxic effects without any significant antibacterial properties. Data from [83] and [84] support the antibacterial performance of zinc-containing chitosan coatings by highlighting the established role of Zn in antimicrobial action. These Zn nanoparticles act mainly through ROS-mediated oxidative damage, along with the destabilization of membranes through electrostatic interactions between the Zn species and bacterial surfaces, as explained in [83]. In [84], it is further enhanced that Zn composite coatings have recognized antibacterial properties, which reduce microbial survival. While Zn-containing coatings did not show inhibition against *S. aureus* in [83], both [83] and [84] indicated interference of zinc species with bacterial surface structures, cellular functions, and limited bacterial adhesion, thus contributing to the overall antibacterial response. The schematic representation of the proposed antibacterial mechanism of chitosan–ZnNP coatings is presented in Figure 2. The major chemical form of applied zinc was zinc oxide [29,85]. The chitosan with incorporated zinc oxide NPs prepared using *Nigella sativa* seed extract demonstrated antimicrobial, antidiabetic, and antioxidant potential [86]. The coating composed of an inner layer of nanoporous TiO_2_ and the outer layer of the chitosan matrix with added ZnONPs showed the antibacterial activity against *E. coli* better than the chitosan coating alone and also inhibited biofilm formation and bioactivity [85]. The positive effects were attributed to the release of Zn^2+^ ions. These ions destabilize the bacterial cell membrane, leading to structural damage and metabolic inhibition. Additionally, the presence of ZnO promotes the generation of reactive oxygen species (ROS), further enhancing the bactericidal effect. As a result, the coating also effectively reduces bacterial adhesion and biofilm formation (Figure 3). A more complex formula presented in [87] was the composite thin layers based on chitosan with molybdenum disulfide nanosheets and zinc oxide NPs, which showed antibacterial efficiency.

Regarding zinc application in orthopedics, the bone regeneration range and antibacterial efficacy of mesoporous glass NPs were demonstrated when supplemented with 2.5–4% ZnO and loaded with curcumin [88]. In [24], 5 g/L of ZnO, together with amikacin, was added to the MAO bath for forming calcium phosphate coating by micro-arc oxidation, demonstrating antibacterial properties.

Zinc was sometimes investigated with respect to its potential for cancer killing. As already mentioned [50], silver-doped zinc oxide nanoparticles were not only antibacterial but also effective against liver carcinoma cells.

A chitosan–ZnO mixture was considered for cotton/polyester blended fabrics [89]. Even if such a solution is possible, using titania instead of zinc oxide becomes preferable.

ZnO was also investigated for its anticancer properties. Tamizhselvan et al. [90] reported that acryloyl chitosan-grafted piperazinium polymers employed as supporting agents for preparing the spherical-shaped ZnNPs showed anticancer behavior against lung cancer cell lines. In similar research [91], the ZnONPs encapsulated within chitosan–camphor became effective against *Echinococcus granulosus* due to suppressing oxidative stress, inflammation, and DNA damage.

As for Ag-implemented chitosan, ZnO-based particles can also be applied in food technology. In a recent study [92], garlic extract-loaded ZnONP–chitosan–PVA nanocomposite film was used as the packaging material to extend the shelf-life of fish fillets. Also, shelf-life extension was observed for grapes due to the chitosan coating reinforced with ZnONPs containing phytocompounds from lemon pomace [93]. As mentioned earlier [72], chitosan coatings with included ZnO and Ag were effective against *Shewanella putrefaciens* and *Pseudomonas aeruginosa* appearing in sea products. The Ti/ZnO/SiOx/chitosan coatings can also be used for food preservation [94]

ZnSe nanoparticles anchored on mesoporous carbon prepared from zinc-based metal organic framework (MOF) and chitosan made it possible to efficiently catalyse the hydrogenation of nitroarenes [95].

## 5. Other Chitosan–Nanometal Coatings and Composite Materials

Few attempts have been made to develop AuNPs added to chitosan. As shown in [96], gold nanoparticles deposited on baicalein/chitosan-modified zinc oxide nanoparticles in preparation of pyranopyrimidines inhibited gastrointestinal stromal tumors. In [83], the presence of gold caused significant hemolysis, presumably due to the specific and not yet fully understood interactions between gold and blood cells. Also, gold initiated cytotoxicity and antibacterial protection. The incorporation of AuNPs into the electrodeposited chitosan coatings induces antibacterial activity towards *Staphylococcus aureus*. The observed effect is apparently linked with the role of nanoparticle–cell interface interactions, while ion release is excluded, as Au exhibits negligible dissolution under physiological conditions. AuNPs interfere with cellular processes, causing a decrease in the metabolic activity of bacteria and an inhibition of proliferation. Bartmański et al. [83] report that Au-containing coatings exhibit less bacterial adhesion in comparison with the unmodified chitosan matrix; therefore, surface modification by AuNPs changes the bacterial–surface interaction. This anti-adhesive effect, combined with suppressed metabolic function, accounts for the antimicrobial behavior of chitosan–Au coatings. The schematic representation of the proposed antibacterial mechanism of chitosan–AuNP coatings is presented in Figure 2. Platinum was only mentioned as a possible antibacterial element [29]. Fascinating osteoblast compatibility and antibacterial activity of HA-Ta_2_O_5_ composite coating deposited by plasma electrolytic oxidation [97]. The effects of silver (Ag), selenium (Se), and chitosan (chit) additives on the antibacterial activity and cell viability (*Saos-2* osteoblast cells) of NiTi were investigated in [53]. The Ag additive to bioceramic coatings significantly increased antibacterial properties but caused a decrease in cell viability. However, although Se–chit additives did not have a significant effect on antibacterial properties (*p* < 0.05), they increased cell viability (*Saos-2* osteoblast cells). In other research for this element, anti-*H. Pylori* activity of chitosan–pirydine chloride Schiff-base possessed the highest biological activity in our investigation, as well as cytotoxicity against colon cancer cell lines [98]. In [56], research was conducted on the chitosan/gelatin/Ag-Mn-doped mesoporous bioactive glass NPs on PEEK/bioactive EPD made on 316 L stainless steel. The addition of biologically active Mn and Ag and chitosan showed a strong bacterial growth effect with no toxic effect on bioactivity. As shown in [99], gallium-modified chitosan/poly(acrylic acid) bilayer coatings improved titanium implant performances against MG63 osteoblast-like cells. Titanium in the form of an intermetallic phase was frequently used to create the bacteriocidal materials. Min et al. [100] proposed the sol-gel synthesized TiO_2_–chitosan nanocomposite as an antibacterial coating for orthopedic implants (the titanium alloy Ti6Al4V). Montaser et al. [101] reported the antimicrobial activity of Schiff-based chitosan and salicylaldehyde/TiO_2_ nanocomposite membrane. Cisternas et al. [102] described phospholipid-supported chitosan–titanium nitride coatings produced by plasma immersion ion implantation as exhibiting clear antibacterial activity.

## 6. Assessment of Properties and Applications of Chitosan–Nanometals

The above review gives evidence that chitosan coatings with implemented metallic nanoparticles, or more seldom their ions, oxides, and sometimes other compounds, were the focus of several investigations. Even though its number is surprisingly limited, such material solutions seem highly interesting for several purposes.

The first purpose is the easy fabrication of such coatings or, not so often, composites. For coatings, the most plausible method is constant direct or pulse electrophoretic deposition. Also, some chemical methods, such as layer-by-layer technique, solution casting, dip and spin coating, and electrochemical techniques such as MAO (micro-arc oxidation), can be considered important and worth further development.

Several properties of the discussed material solutions, and especially the coatings, are promising. In particular, as shown below, the biological properties are the most important factors for metal-implemented chitosan-based coatings. Additionally, the mechanical behavior of such coatings is very likely better than that of simple chitosan coatings.

The most interesting properties here considered are antibacterial and antivirus capacities and bioactivity in chemical and biological contexts. These aspects are discussed below in light of the dual challenge posed by chitosan–metal nanoparticles: their effectiveness in bacterial eradication and their influence on osteoblast behavior. The characteristics of chitosan-based antimicrobial and anticancer composites and coatings are summarized in Table 1, which compiles recent studies addressing both their bactericidal performance and biocompatibility. Nevertheless, the most intriguing and unrecognized issue remains the competition between processes affecting antibacterial properties and cytotoxicity. It is not surprising as both phenomena are strictly related to the capacity of nanometals to kill the cells, such as bacteria, viruses, or cancer, or other human/animal cells, e.g., osteoblasts. Both processes are related to many factors, with the nanometal content considered the most important.

The elements taken into account here are hazardous in high concentrations. In an earlier review [107], nanosilver was found to be cytotoxic to several different cell lines, including mouse fibroblast, monocytes, rat liver cells, male mouse germline cells, human lung fibroblast cells, and human. According to Agnihotri et al. [108], the incorporation of Ag, Au, Ti, Zn, Cu, and ZrNPs in dental materials enhanced antimicrobial, mechanical, and regenerative properties, but toxicological consequences can appear, expressed by cytotoxicity, genotoxicity, or at least inflammation. This issue remains highly controversial. According to Zaimoglu et al. [109], nanometals such as Ag, Zn, and Cu demonstrate both antibacterial efficacy and cytotoxicity versus human cells. The main mechanisms include the depolarization and perturbation of the membrane integrity, K^+^ ion leakage, osmotic collapse, DNA and ribosome denaturation, and production of ROS. Silver ions released after nanoparticle degradation depolarize mitochondrial membranes and damage mitochondrial DNA, causing ROS production as electron transport chain by-products. Nanozinc triggers ROS production, resulting in bacteria killing, DNA damage, genotoxicity, and apoptosis of human cells. Nanocopper again has shared mechanisms of bactericidal activity and human cell toxicity. The difference between all these elements is in the concentration that triggers both processes, and their intensity and time dependence. By [28], the cytotoxicity of nanometals at higher doses is dangerous, as Ag^+^ ions can bind to thiol groups of proteins and enzymes, disrupt respiratory chains, and cause membrane permeability. They also penetrate cells, interacting with DNA and negatively affecting its replication. Likewise, Cu nanostructures demonstrate Fenton-like interactions, causing the appearance of hydroxyl radicals and oxidative disruption. AuNPs, though less intrinsically toxic, are exceptional carriers for antipathogens. According to [109], and considering both nanoparticles and free ionic silver, copper, and zinc, injected intraperitoneally, damage to the brain for high doses of nanosilver and nanometal combinations, damage to the liver, and, in particular, damage to the kidneys also appeared. The highest metal concentration was observed between the 30th and 60th day.

At high concentrations, the results observed in in vivo studies of different nanometals are astonishing. Guo et al. [107] reviewed the available data on the cytotoxicity of nanosilver toward mammalian cells and confirmed its occurrence. In an early work, Hadrup et al. [110] found that orally administered silver was absorbed in a range of 0.4–18% in mammals, with a human value of 18%. The highest levels were observed in the intestine and stomach. By [111], as observed in inhalation studies, silver and gold were retained in the lung for >2000 and >672 h, respectively; copper content initially increased in the lung and then decreased to the initial value at ~500 h, and zinc presence was more observed in the lungs, but only after short-term exposure to zinc oxide. In blood, the presence of silver initially increased and then declined in ~200 h. Gold concentration was elevated for 672 h for smaller nanoparticles, 4–13 nm, but not for greater NPs, 20–105 nm. Silver level increased in the liver and the spleen, gold in the spleen and kidney, and both in the brain and olfactory bulb. Interestingly, even though no significant differences were apparent in the distribution of the four nanomaterials, their levels were elevated for a longer time for silver and gold.

The critical aspects are the size of the nanoparticle, with a critical boundary value between strong and weak (or not at all) toxicity of about 10–20 nm, sometimes more, depending on the body organ. Even a small increase in size may significantly decrease the toxic effects in clinical attempts [112,113]. Another approach to diminish toxicity is to reduce hydrophobicity. Metals, both as free ions and nanometal formulations, interact with body fluids. In these conditions, when in the blood, a corona is formed composed of dynamically changing proteins approaching the nanometals and enhancing nanometal toxicity. For two reasons, the capture of nanometals is more likely by the phagocytic cells, and the protein corona enhances the delivery of metal ions from the exterior to the interior of the cells according to the “Trojan horse” mechanism. Thus, reducing the hydrophobicity can essentially improve biosafety [114].

However, until the nanoparticles remain at stable positions at the titanium surface, they only slowly dissolve in SBF, and they are not in the blood. The serious problem appears a long time after implantation due to the degradation of titanium implants. Until then, the silver, copper, zinc, and other ions are important, and their nanoparticles are the only source of ions. The release rates and local concentrations of various metallic ions depend in such cases on a variety of factors, including the exposed surface area of nanoparticles, pH, and temperature, which determine the thermodynamic possibility and kinetic reaction rate, among others. Cytotoxicity depends critically on the release rates.

The biocompatibility is then strictly related to a local concentration of metallic ions, which do not negatively affect the living body’s behavior, and also whether such an element is present. Cytotoxicity is a phenomenon that appears when the local concentration exceeds a certain limit, provoking the defensive reaction of the body. Quite early, the effects induced by particulate silver were attributed to silver ions released from the particle surface [107,110]. Nagime et al. [115] noticed that the major difference between the AgNPs and AuNPs was claimed to be in the occurring processes: AgNPs release Ag ions that bind to thiol groups in vital enzymes, disrupting bacterial metabolism and deactivating essential proteins, and AuNPs exhibit antibacterial properties via their interference with the bacterial electron transport chain, reducing ATP production and causing energy starvation. Copper and zinc belong to biometals, even if their excess, like for other biometals, is not recommended. Cu and Fe are well-known functional components in several cellular and metabolic processes; however, in high concentrations, they can undesirably affect biological phenomena [116]. Normally, the cytotoxic level of nanosilver or silver ions is much higher than the antibacterial level. However, long-term exposure to low concentrations of nanosilver can induce toxicity. On the other hand, this cytotoxicity at high doses is utilized [117] for Ag, Au, Fe, and CuNPs in medicine. Silver nanoparticles show strong antimicrobial potential, including activity against multidrug-resistant pathogens, while gold nanoparticles stand out in cancer therapy due to their excellent biocompatibility. Iron and copper nanoparticles are also of interest in antimicrobial therapy.

The release rates, and in particular, the burst time release and sustained time release, of different metals from their nanoparticles (or oxide nanoparticles) can then only be roughly estimated, and such data are scarce. For example, in [118], citrate-coated AgNPs form agglomerates in alkaline simulated body fluids, reducing the surface area and dissolution rate, to a degree dependent on pH and ionic strength of the liquid. That means that silver nanoparticles in acidic media possessing high ionic strength will demonstrate short-term effects similar to those of ions. Tomić et al. [119] proved that the release profiles of silver, iron, and zinc inorganic compounds from hydrogels showed an initial rapid release followed by a slower step.

The composite materials and coatings were developed for different purposes, but among them, a highly important need appeared for a decrease in burst release time, time to achieve the sustained release, maintaining the maximum concentration below the safe limit, and maintaining the antibacterial effectiveness for a long time. Such a compromise is not always obtained, e.g., in [120], alginate-capped AgNPs retained promising antibacterial activity for 1% alginate, but they became toxic to mammalian cells at the same concentration. Moreover, all the tested mammalian cells disclosed sensitivity at much lower silver doses compared to bacteria. For example, Foss et al. [121] reported that the incorporation of chitosan on a titanium surface greatly encouraged osteoblast adhesion behaviors (including cell initial attachment, adhesion, and spreading) while significantly decreasing bacteria attachment. Similar but not the same opinions can be found in the literature. Covato et al. [122] proved that the osteoblasts win the race for the surface on DNA polyelectrolyte multilayer coatings against *S. epidermidis* but not against *S. aureus*. Chromium-doped hydroxyapatite in a chitosan matrix coating demonstrated an important bioactivity at no cytotoxicity, with simultaneous antibacterial efficiency against *Pseudomonas aeruginosa*, *Escherichia coli*, and *Staphylococcus aureus* [106]. A chitosan–lauric acid conjugate was successfully immobilized onto the surfaces of Ti, which improved the cell adhesion and viability, alkaline phosphatase activity, and mineralization rate. Simultaneously, inhibition against *Staphylococcus aureus* and *Pseudomonas aeruginosa* was observed [123]. Then, the addition of ZnO to chitosan imparted antibacterial properties, and did not negatively influence biocompatibility and osteogenic differentiation of cells in chitosan-gelatin hydrogel [105]. Ultrasmall zinc oxide nanoparticle-reinforced chitosan–fucoidan scaffolds possessed significant antibacterial activity and high osteogenesis rate [104]. Qi et al. [124] tested the biocompatibility in vitro by cell live/dead staining, cell viability assay, and cell proliferation for PEK in foam form with Ag nanoparticles. The live/dead staining demonstrated that cells could survive well on the surfaces with and without silver after 24 h of incubation. For the cell viability assay after 24 h, both surfaces maintained cell viability over 90%. Cytotoxicity testing of chitosan–thyme extract–nanosilver nanocomposites showed that lower Ag concentrations were preferable for maintaining cell viability [125]. According to Guo et al. [126], there was an inconsistency in the results, namely, when nanosilver was added to the glass ionomer cement, either cellular damage by the production of reactive oxygen species was observed or nanosilver-modified cement did not influence cell viability. In other research [127], bovine lactoperoxidase (LP) and lactoferrin (LF) were found to be suitable proteins when coated by or adsorbed to nanocopper and nanoiron. The authors developed composites of LP–CNPs and LF–FNPs that had a spherical shape with an average nanosize of about 21 nm. In other work [128], the AgSiO_2_ coatings were investigated, demonstrating that the cells could grow and proliferate on all coatings. However, when compared to the pure SiO_2_ coating, the highest 50 wt. pct. Ag coating showed a significantly lower difference in cell number, especially after 12 days, probably due to the Ag^+^ ion release. Controlled Ag release can also be achieved with the hybrids composed of vaterite CaCO_3_ and AgNPs, which showed a burst release at pH 5 and no AgNPs being released at pH 7.4 and 9.0 [129]. In a recent review [130], the incorporation of various nanoparticles into polymethyl methacrylate (PMMA)-based resins in in vivo studies conducted on humans or animals with nanoparticles of silver, titanium dioxide, copper, and gold confirmed the absence of adverse effects in cell cultures and human volunteers. The question may be born for what purpose different nanometals are sometimes applied together, either as different layers in multicoating or as a co-precipitated coating. Silver and copper are exclusively added as strong antibacterial elements in the coating. Elementary zinc can also play a similar role, but it is the most often added in the form of ZnO, and this ceramic can substantially improve the strength, toughness, and adhesion of the coating to titanium, similar to, e.g., silica or titania (even such investigations are scarce). An addition of two or even more (not found so far) antibacterial nanometals allows for a decrease in the level of each element, thus potentially decreasing the risk of early cytotoxicity. Such synergetic interaction was observed for Ag/ZnO/Gelatin gel particles against *S. aureus* based on the growth inhibition assay and agar dilution method, and the effect was better compared to Ag/Gelatin and ZnO/Gelatin [131]. The well-distributed silver and zinc oxide nanoparticles in the composite gel also showed the highest increase in the mean lifespan by protecting *Caenorhabditis elegans* from infection. In other research [132], the antibacterial activity of Ag–ZnONPs was estimated against *B. subtilis*, *E. coli*, *K. pneumoniae*, *P. aeruginosa*, and *S. aureus*. The maximum zone of inhibition, 24 mm, was observed for *K. pneumoniae*. The effect was attributed to the antibacterial character of ZnO and Ag metals, which could act together on bacterial cells, thus demonstrating the synergetic effect. Behzadnia et al. [133] observed that, in the testing of Ti, Ag/Ti, Zn, Ag/Zn, Ti/Zn, and Ag/Ti/Zn, it was observed that introducing Ag nanoparticles to TiO_2_/ZnO nanocomposites resulted in greater antimicrobial activity.

The properties associated with NPs also need to be carefully adjusted; otherwise, they can also pose a problem in the development of a successful nanoparticulate system. Surface properties, biocompatibility, immunogenic reactions, cytotoxicity, and interaction with cells appear to be huge challenges in the formulation of coatings and composite materials based on chitosan and nanometals. In clinical research, most of the nanoparticulate systems are still in phase I of clinical trials. In particular, the absence of specific regulatory guidelines for the formulation and development of such NP-implemented materials highly complicates the translation of clinically tested nanoparticle systems into marketable products [134]. On the other hand, clinical trials are very expensive and can take many years, especially when long-term toxicity is studied. So, the passage from scientific research on the first TRL (technical readiness level) to the higher TRLs is possible only for some selected technical solutions and requires serious financing. Even after positive clinical results, the allowance for any medical application takes several years. Thus, at first, investigations and theoretical work are still necessary to understand the processes and mechanisms involved in providing desirable properties to tested systems, strictly in achieving biological activity without short-term and long-term toxicity. The appropriate regulatory guidelines should also be recommended to increase the rate of translation from a successful laboratory to a clinically approved nanoparticulate product. It is interesting that despite these challenges, more than 1200 patents on Ag/AuNPs alone were registered in the past five years, and almost 0.2 million research articles related to nanotechnology were published in scientific journals [135].

## 7. Conclusions

Future developments in the area of fabrication of chitosan-based coatings will likely be directed at hybrid, stimuli-responsive fabrication approaches that not only ensure precise spatial control over nanometal distribution but also respond to specific physiological or pathological cues. These include pH/inflammation-responsive chitosan coatings that modulate ion release according to the extent of the local pathology, and greener synthesis methodologies that minimize the environmental impact of nanometal generation. Moreover, tight integration of state-of-the-art characterization with in silico modeling may serve to optimize process parameters towards producing coatings with predefined microstructure, mechanical properties, and release performance.

Chitosan–metal nanocomposite coatings embody one of the most versatile classes of hybrid biomaterials that unite the bio-functionality of chitosan with the antibacterial, catalytic, and bioactive potential of metallic nanoparticles. Among them, chitosan enriched with silver, copper, and zinc nanoparticles has shown the most consistent performance in biomedical and food-related applications. These coatings exhibit pronounced antibacterial and antifungal properties, in some cases showing antiviral activity, while acceptable cytocompatibility is generally well maintained after optimization for nanoparticle concentration and release rate. In addition, they often enhance the mechanical strength, adhesion, and corrosion resistance of the coated substrates.

Despite such promising results, a number of challenges still exist. The mechanisms for the dual antibacterial and cytotoxic properties of metallic nanoparticles themselves are still not thoroughly understood; these depend strongly on particle size, oxidation state, and the local microenvironment. Besides, the reproducibility and scalability of deposition techniques like electrophoretic deposition, layer-by-layer assembly, and chemical reduction are still critical concerns. Further efforts concerning the establishment of standard fabrication parameters and quantification of long-term biological safety should be made in a more systematic way.

The possible cytotoxicity of the nanometals considered here can appear at high doses. The assessment of this risk of such a phenomenon for chitosan–nanometal composite materials and coatings is still difficult and needs further biological tests. Nevertheless, so far, research on such composites with usually applied nanometal contents indicates that it is possible to avoid toxic effects while obtaining sufficient antibacterial protection, in particular, when achieving the slow release rate of nanometals and their moderate highest top levels.

In view of clinical translation, future studies on chitosan–nanometal coatings must focus more on long-term performance. In particular, a systematic coating lifetime, degradation rate, and ion release evolution under simulated physiological conditions has to be performed. The studies not only have to investigate the change in antibacterial activity with time but also assess the long-term biocompatibility of the coatings, including metal species accumulation or depletion and the consequences for osteoblast function and tissue integration. Long-term in vitro models will need to be combined with in vivo studies to establish whether such systems can maintain an optimal balance between antimicrobial efficacy and safety over the entire service life of the implant. Future studies should be directed at the rational design of chitosan–nanometal systems bearing controlled ion release kinetics, improved biocompatibility, and multifunctionality tailored for specific applications, either biomedical or technological.

## Figures and Tables

**Table 1:** Characteristics of chitosan-based antibiocidal and anticancer composite materials and coatings.

Base Composition	Metal Nanoparticles	Physical Form	Fabrication	Reference
Chitosan–DHBA	Ag	Coating	EPD	[43]
Chitosan	Ag	Composite	Hydrothermal synthesis	[44]
Carboxyle chitosan	Ag	Composite	Synthesis	[45]
Chitosan	Ag	Composite	Sonochemical synthesis	[49]
Chitosan–PVA	Ag	Coating	Spread casting	[51]
Chitosan	Ag	Coating	Electrodeposition	[52]
Chitosan	Ag	Composite	Synthesis	[54]
Chitosan	Ag	Coating	Drying	[30]
Carboxyle chitosan	Ag	Composite	Freeze drying	[55]
Carboxyle chitosan–ginger oil	Ag	Composite	Synthesis	[58]
Chitosan	Ag	Composite	Synthesis	[59]
Chitosan–rGO	Ag	Coating	Solution casting	[103]
Chitosan–PVA–BG	Ag	Coating	EPD	[61]
Chitosan–TPP–nHAp	Ag	Hydrogel	Synthesis	[62]
Chitosan–fluor	Ag	Solution	Synthesis	[63]
Chitosan–PPy–lignin	Ag	Hydrogel	Synthesis	[64]
Chitosan–PVA	Ag	Coating	Drying	[65]
Chitosan–silk deferoxamine	Ag	Composite	Freeze drying	[66]
Chitosan–SiO_2_	Ag	Coating	Electrospinning	[67]
Chitosan	Ag	Coating	EDP	[41,42]
Chitosan–PEG	Ag	Coating	EPD	[68]
Chitosan–PVA	Ag–ZnO	Coating	Drying	[50]
Chitosan	Ag–Se	Coating	Sol-gel	[53]
Chitosan–gelatine–BG	Ag, Mn	Coating	EPD	[56]
Chitosan	Cu	Coating	Layer-by-layer	[73]
Chitosan	Cu	Coating	EDP	[42,74]
Chitosan	CuO	Coating	Spraying	[77]
Si–chitosan–cyclodextrin–rosmarinic acid	Zn	Coating	Immersion	[79]
CaP–chitosan	Zn	Coating	MAO	[24]
Chitosan	Zn	Coating	EDP	[83,84]
Porphyrin–chit chitosan	Zn(Pd)–Ag	Hydrogel	Synthesis	[46]
Chitosan–cellulose–HIA	Zn (carbonate NPs)	Composite	Synthesis	[81]
Chitosan	Zn (La-Zn ferrite)	Composite	Synthesis	[82]
Chitosan	Zn (oxide)	Composite	Synthesis	[86]
Chitosan	Zn (oxide), Mo (sulfite)	Coating	Mixing	[87]
Chitosan	Zn (oxide)	Solution	Synthesis	[89]
Acryloyl chitosan–grafted piperazinium	Zn (oxide)	Composite	Synthesis	[90]
Chitosan–camphor	Zn (oxide)	Gel	Synthesis	[91]
Chitosan–fudoidan	Zn (oxide)	Scaffold	Synthesis	[104]
Chitosan–gelatin	Zn (oxide)	Composite	Freeze drying	[105]
Chitosan	Au	Coating	EDP	[83]
Baicalein–chitosan	Au, Zn (oxide)	Composite	Synthesis	[96]
Chitosan–poly(acrylic acid)	Ga	Coating	Electrodeposition	[99]
Chitosan	Ti (oxide)	Coating	Sol-gel	[100]
Chitosan–phospholipid	Ti (nitride)	Coating	Plasma immersion/PVD	[102]
Chitosan–salicylaldehyde	Ti (oxide)	Membrane	Casting	[101]
Chitosan–HAp	Cr (in HAp)	Coating	Dip coating	[106]

DHBA—3,4-dihydroxybenzaldehyde (DHBA), PPy—Polyvinylpyrrolidone, rGO—reduced graphene oxide, HIA—hyaluronic acid.

## Data Availability

No new data were created or analyzed in this study. Data sharing is not applicable to this article.
